# P02.87. Ayurvedic management of Ulcerative Colitis: a non-randomized, observational cinical study

**DOI:** 10.1186/1472-6882-12-S1-P143

**Published:** 2012-06-12

**Authors:** P Kalapi, P Manish, S Namrata, K Christian, S Gupta

**Affiliations:** 1 J.S.Ayurved College, Nadiad, Gujarat, India; 2 Charité Medical University, Berlin, Germany

## Purpose

Ulcerative Colitis (UC) is a chronic inflammatory bowel disease with a relapsing and remitting course. The prolonged use of conventional standard drugs often produces mild to severe side effects and may eventually result in drug resistance. Hence, there is a need for effective and safe complementary treatment options. In Ayurveda various treatment modalities for chronic inflammatory bowel diseases are described and used successfully.

## Methods

A total of 50 patients with clinical features of UC and a confirmed endoscopic diagnosis of UC were included. All patients received complex Ayurvedic treatment for 4 weeks. Treatment included oral administration of herbal drugs (Holarrhena antidysenterica, Ficus glomerata, Cyperus rotundus, Mesua ferrea and Symplocos racemosa), recto-colonic administration of Ficus glomerata and Ayurvedic dietary advice (avoidance of spicy, sour, fried, hot and heavy food items). Patients were assessed for changes in clinical features and laboratory investigations including haemoglobin-%, erythrocyte sedimentation rate (ESR), presence of occult blood, red blood cell count and pus cell count in stool. Data were analyzed statistically by using student’s t-test.

## Results

All 50 patients completed the four-week Ayurvedic treatment. Results show highly significant (p<0.001) reduction of the frequency of bowl-movements (77.4±4.2%) and blood presence in stool (90.3±0.8%). The reduction in the requirement of conventional standard drugs was also highly significant (steroids 100%, sulfasalzine 80.26±0.9%). UC-associated symptoms like abdominal pain, weakness and weight loss were relieved significantly. Laboratory value improvement in haemoglobin-% (14.4±0.8%), ESR (39±10.2%), erythrocytes (91.7±1.1%) and pus cells (83.7±0.9%) in stool were also found statistically highly significant. Moreover, no treatment-related adverse events could be observed.

## Conclusion

Ayurvedic treatment of ulcerative colitis and other chronic inflammatory bowel diseases is effective in reducing disease symptoms and disease relapses and can reduce the need of conventional standard drugs. Moreover, the treatment is safe, inexpensive, easy to handle and well tolerated by the patients.

